# Molecular Insights into IAHSP: Influence of the R1611W Mutation on the VPS9 Domain of Alsin

**DOI:** 10.1021/acsomega.5c05926

**Published:** 2025-11-05

**Authors:** Marcello Miceli, Cécile Exertier, Elena Gugole, Beatrice Vallone, Marco Agostino Deriu

**Affiliations:** † Polito^BIO^Med Lab, Department of Mechanical and Aerospace Engineering, 19032 Politecnico di Torino, Torino 10129, Italy; ‡ Department of Life Sciences, Università Degli Studi di Modena E Reggio Emilia, Via Campi 103, Modena 41125, Italy; § Institute of Molecular Biology and Pathology, Italian National Research Council (ibpm-Cnr), c/o Department Biochemical Sciences, Sapienza University of Rome, Ed. CU027, P.le A.Moro 5, Rome 00185, Italy; ∥ Department of Biochemical Sciences “ALESSANDRO ROSSI FANELLI”, Università La Sapienza, Rome 00185, Italy

## Abstract

Mutations of the alsin protein have been linked to infantile-onset ascending hereditary spastic paraplegia (IAHSP), a rare neurodegenerative disease. More precisely, the pathological R1611W mutation has been identified in the Vacuolar Protein Sorting 9 (VPS9) domain, which acts as a guanine nucleotide exchange factor (GEF) for Rab5. This mutation results in the expression of tryptophan instead of arginine that alters the oligomeric state of alsin and its GEF functions. Insights into the conformational structure of the wild-type or mutant VPS9 domain may help elucidate the mechanisms involved in the onset of the disease. In this study, we combined *in vitro* and *in silico* approaches to elucidate the structure and understand the effects of the R1611W mutation on the isolated VPS9 domain of alsin. This mutation induces conformational changes that alter the local structure of the protein and its ability to oligomerize. This study lays the groundwork for understanding how R1611W alters the VPS9 domain function.

## Graphical abstract

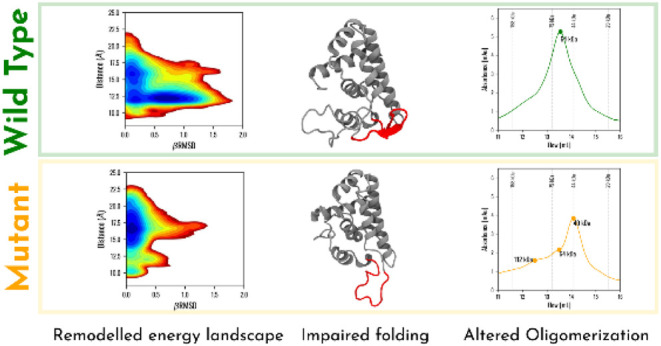

## 1 Introduction

Infantile-onset ascending hereditary spastic paralysis (IAHSP) is a rare neurodegenerative disease (prevalence <1:100000).^1^ Symptoms typically emerge in early childhood, with variability among individuals. They usually present as spasticity that initially affects the lower limbs and, over the following decade, progresses to involve the upper limbs, ultimately leading to quadriplegia.^2–4^ It is an autosomal recessive pathology characterized by the retrograde degeneration of upper motor neurons of the pyramidal tract due to a mutation in the *ALS2* gene at locus 2q33.1, which encodes a protein mostly expressed in the cerebellum and the spinal cord, namely alsin.^5–7^ Alsin is a 1657-residue-long protein endowed with guanine nucleotide exchange factor (GEF) activity on small guanosine triphosphatases (GTPases). It is composed of four independent structured domains identified by sequence homology (Figure S1): (1) the RLD, which is homologous to the regulator of chromosome condensation 1-protein (RCC1) and is important for subcellular localization and endosomal association;^8,9^ (2) the Dbl Homology and Pleckstrin Homology (DH/PH) domain; (3) the eight consecutive membrane occupation recognition nexus (MORN) motifs; and (4) the Vacuolar Protein Sorting 9 (VPS9) domain, which likely works synergistically to allow alsin homo-oligomerization and to carry out GEF activity on the Rab5 GTPases.^10^

IAHSP is associated with various types of mutations in alsin,^1^ such as missense mutations or truncations. In addition, mutations in different alsin-structured domains have been associated with this pathology.^1^ A molecular event crucial to the proper functioning of alsin is the formation of a homotetramer.^11^ It has been observed that mutations can alter the protein’s oligomeric state, inducing the formation of other potentially pathological oligomers, such as dimers, trimers, as well as pentamers or hexamers.^11^ Obtaining structural and functional insights into the physiological behavior of alsin and its pathological malfunction is a crucial step in understanding IAHSP pathology and the development of treatment. This work is part of a set of investigations in which we focused on the different alsin domains and their implications in the biological function of the whole protein.^9,12,13^ We previously focused on the RLD selectivity for phosphoinositide lipids,^9^ analyzed the conformational dynamics of the DH/PH domain and how this is influenced by the interaction with Rac1,^12^ and investigated the role of the MORN domain as a mediator of homophilic interaction.^13^

This present work investigates the missense pathological mutation of arginine 1611 into tryptophan (R1611W),^14^ which occurs in the C-terminal VPS9 domain region of alsin. An *in vitro* study has shown that the R1611W mutation alters the alsin oligomerization equilibrium toward a trimeric arrangement.^11^ To investigate how the R1611W mutation affects the local conformational dynamics of the VPS9 domain in the absence of any experimental structural data on human alsin, we carried out a structural analysis of the VPS9 domain through integrated *in vitro* and computational methods. An experimental protocol was developed to express and purify the wild-type (WT) and R1611W mutant human alsin VPS9 domains. These VPS9 proteins were characterized by size exclusion chromatography (SEC) to determine the oligomeric arrangements of VPS9 homomers and by circular dichroism (CD) to characterize their secondary structure, a step that may clarify how structural changes impact functional GEF activity relevant to the disease. The *in silico* approach explores the conformational dynamics of the monomeric VPS9 at atomistic resolution. Since the 3D experimental VPS9 structure is still lacking, we used the AlphaFold V2 ^15^ algorithm to predict the WT and mutated VPS9 structures. These two models were dynamically characterized by employing extensive force field-based molecular dynamics simulations to gain insight into the distribution of secondary structure and the network of noncovalent interactions, with a particular focus on those involving the mutant residue. Finally, we performed temperature-replica exchange molecular dynamics to investigate the conformational folding of VPS9 energetically and to understand how the mutation reshapes the energy landscape and compromises local folding stability. Notably, we observed that the mutation leads to the local misfolding of the protein.

## 2 Materials and Methods

### 2.1 Expression and Purification

The human alsin VPS9 WT (C1551S/C1558S/C1647S) gene construct (UNIPROT Q96Q42 1513–1657) was synthesized and subcloned into a pOPINM vector by GenScript. Later, the gene was subcloned into a pET28b vector as follows: the VPS9 gene (insert) was extracted from the pOPINM by enzymatic digestion using NdeI and *Eco*RI restriction enzymes. The same enzymes were used to digest the pET28b plasmid. The insert was then amplified by PCR using the TGCCGCGCGGCAGCCATATGAAGCAGCCGG and GTGCGGCCGCAAGCTTGTCGACGGAGCTCGAATTCTTAGTTCAGTTTTTCACGTTG primers and the OneTaq Quick-Load Master Mix 2X (New England Biolabs). Successful ligation was achieved by Gibson Assembly (New England Biolabs) and checked by sequencing (Eurofins). The R1611W mutation was inserted using the QuikChange Lightning Site-Directed Mutagenesis kit (Agilent). VPS9 WT and the R1611W mutant were expressed in *E. coli* BL21 (DE3). Cells were grown at 37 °C until the optical density (OD) reached 0.6–0.8. The cultures were subsequently induced using 1 mM IPTG and further incubated at 37 °C for 3 h before harvest.

Cells were resuspended in 50 mM Tris (pH 8.5), 300 mM NaCl, supplemented with complete antiprotease cocktail, DNase, RNase, and lysozyme at 4 °C for 1 h. Cells were sonicated and centrifuged. Pellets were washed several times in 50 mM Tris (pH 7.5), 100 mM NaCl, 0.5% Triton X-100, and 1 mM DTT and once in 100 mM Tris (pH 7.5), 150 mM NaCl, and 1 mM EDTA. Inclusion bodies were finally resuspended in 8 M urea, 50 mM Tris (pH 7.5), 0.1 mM EDTA, and 0.1 mM DTT before purification by immobilized metal affinity chromatography (HisTrap columns, Cytiva, USA). Protein constructs were eluted using 500 mM imidazole. After concentration and buffer exchange, proteins were diluted drop by drop in 3 M urea, 50 mM Tris (pH 7.5), 150 mM NaCl, 0.1 mM EDTA, 0.5 M l-arginine, 0.2 mM MgCl_2_, and 1% glycerol (final protein:buffer ratio of 1:70 ratio) and incubated at 4 °C overnight. The protein was dialyzed against 50 mM Tris (pH 7.5), 150 mM NaCl, 1% glycerol, and 50 mM urea, and later against 50 mM Tris (pH 7.5), 150 mM NaCl, and 1% glycerol. Proteins were then concentrated by loading onto a HisTrap column (Cytiva, USA) and eluted using 600 mM imidazole. The buffer was exchanged into 50 mM potassium phosphate (pH 8.0) and 150 mM NaCl. Sample purity was assessed by SDS-PAGE.

### 2.2 Circular Dichroism

To assess the refolding of the sample and evaluate the secondary structure content, circular dichroism (CD) spectroscopy experiments were performed on 0.62 mg/mL VPS9 WT and 0.53 mg/mL VPS R1611W in 50 mM potassium phosphate (pH 8.0) and 150 mM NaCl, using a 0.1 cm path length quartz cuvette (Hellma, Plainview, NY) on a JASCO J-815 spectropolarimeter equipped with a Jasco CDF 426*S*/15 programmable Peltier element (Jasco, Easton, MD, USA) to ensure precisely controlled temperature.

Far-UV (200–250 nm) CD data were recorded. For each sample, three independent acquisitions were collected and averaged, and this process was repeated for three independent purifications of both the wild-type and mutant domains. The spectra were then converted into mean residue ellipticity (MRE) according to eq 1, baseline-corrected and averaged.

1 $$\left[\Theta \right]=\frac{mdeg\times M_{w}}{10\times L\times C}$$

where [θ] is the CD in MRE, mdeg is the CD in milli degrees, *L* is the cuvette path length, *C* is the sample concentration in mg/mL, and *M*_W_ is the molecular weight in kDa.

After the averaging procedure, a Savitzky–Golay filter of order three with a window size of 9 samples was applied to smooth each spectrum. Secondary structures were predicted from the spectra using the SESCA suite.^16,17^ The best basis set was chosen by selecting the spectra with the lowest root mean squared deviation (RMSD), after spectrum scaling, between the experimental CD and the predicted spectrum. The secondary structure prediction was then grouped into three classes, which represent the α-helix (α-helix), beta-strand (β-strand) content, and coil or unstructured regions (coil).

### 2.3 Size Exclusion Chromatography

Size exclusion chromatography (SEC) was performed by using a Superdex 200 Increase 10/300 GL column (Cytiva, USA) coupled to an HPLC system equipped with a UV–vis absorbance detector (Azura System, Knauer–Berlin, Germany). The measurement was carried out on both the wild-type and mutant for one of the three independent purification samples since the reproducibility of the purification procedure was confirmed by the consistent CD spectra obtained from all three independent purifications. The column was equilibrated with 50 mM potassium phosphate, pH 8.0, and 150 mM NaCl. A total of 310 μg of VPS9 WT and 270 μg of VPS9 R1611W were injected onto the column and eluted at a flow rate of 0.5 mL/min in isocratic mode. The elution profile was monitored at 280 nm at room temperature.

### 2.4 Molecular Modeling of Alsin VPS9 Domain

A fully atomistic molecular model for the VPS9 domain of alsin has been built through artificial intelligence-based protein structure prediction. Models were built and compared to experimental data to provide insights into, at an atomistic scale, the structural effects of the R1611W substitution in the VPS9 domain. For this reason, two systems were built and simulated through force field-based molecular dynamics (MD) and Temperature-Replica Exchange MD (T-REMD), namely the wild-type VPS9 domain (VPS9^WT^) and the mutant (VPS9^R1611W^).

### 2.5 VPS9 WT and R1611W Molecular Models

Human alsin sequences were retrieved from the UniProt database (ID: Q69Q42), and the residues corresponding to the predicted VPS9 domain (amino acids 1513–1657) were extracted and numbered according to the whole alsin sequence. To compare the results to experimental data, the sequences (MGSSHHHHHHSSGLVPRGSHM) of the His-tag and thrombin binding site were added to the VPS9 sequence. From now on, we refer to the His-tag/thrombin site as the purification tag. The mutant sequence R1611W was obtained by substituting the arginine at position 1611 with tryptophan in the primary sequence. Then the ColabFold platform^18^ was employed to predict the three-dimensional structure of VPS9^WT^ and VPS9^R1611W^. Model quality was assessed using the average predicted local distance difference test (pLDDT), retrieved as a result of the prediction procedure, and the Z-score obtained through the ProSA-web server,^19^ while stereochemical quality was assessed by evaluating the Ramachandran plot using the MOE platform. Moreover, to assess the influence of the purification tag on the prediction of the three-dimensional model, the VPS9^WT^ structure was compared to the whole alsin structure (https://alphafold.ebi.ac.uk/entry/Q96Q42) retrieved from the AlphaFold database,^20^ in terms of RMSD. The nonbonded interactions between amino acid 1611 and the rest of the protein in both models (i.e., VPS9^WT^ and VPS9^R1611W^) were analyzed using the MOE software.^21^

### 2.6 System Setup and Molecular Dynamics

Atomic positions for the VPS9^WT^ and VPS9^R1611W^ domains were obtained from the output of the molecular model-building procedure. The CHARMM36m force field^22^ was employed to build the topology of the system. The protein was protonated according to pdb 2pqr^23^ prediction at pH 8. Then, the protein was inserted into a cubic box with periodic boundary conditions defined, setting a minimum distance of 1 nm between the protein and the box edge. Then, it was solvated in explicit TIP3P water, and an appropriate number of Na^+^ and Cl^–^ were added to reach a physiological concentration of 0.15 M and to neutralize the charge. Energy minimization was performed using the steepest descent method for 2000 steps. An initial simulation of 500 ps in the NVT ensemble and a subsequent simulation of 500 ps in the NPT ensemble were carried out, both under positional restraints of the α-carbons. The NVT simulation was performed at a reference temperature of 298.15 K (0.1 ps) using the modified Berendsen thermostat.^24^ The NPT simulation was carried out at 1.0 bar using a Berendsen barostat with isotropic coupling (1.0 ps). Finally, an MD simulation in the NPT ensemble was conducted for 2 μs. The equation of motion was integrated using the leapfrog algorithm by using a time step of 2 fs. Electrostatic interactions were treated with the particle mesh Ewald method, with a short-range cutoff of 1.2 nm. Van der Waals interactions were treated with a cutoff of 1.2 nm and a switching of the potential starting at 1.0 nm. The simulation engine employed was GROMACS 2022.^25^ Three replicas were performed for each condition, resulting in a total of 6 μs of simulation for each condition.

### 2.7 Temperature Replica Exchange Molecular Dynamics Setup

After the production MD simulations, configurations from the MD were extracted to perform temperature replica exchange MD (T-REMD) simulations at increasing temperatures to better explore the conformational space of VPS9^WT^ and VPS9^R1611W^. The T-REMD simulations were performed through 152 replicas spanning temperatures between 298.15 and 450 K; details of the temperatures are reported in Table S1. The Patriksson and van der Spoel temperature predictor^26^ was employed to define the number of replicas and the temperatures to guarantee an average exchange probability above 30%. Each replica was simulated for 200 ns, with exchanges between replicas attempted every 400 fs, resulting in a total of 30.4 μs of simulation. The force fields, electrostatic interaction treatment, and details about the equation of motion integration were the same as those used in the classical MD simulation. The convergence of the T-REMD simulation was assessed by observing the average exchange rate and the superposition of the energy between the replicas. Moreover, the exploration of the temperatures has been reported for the replica of interest at 298.15 K in the Supporting Material.

### 2.8 Conformational Analysis

After the MD simulations, analyses to assess the stability of the simulation were performed using RMSD calculations. RMSD was evaluated on the core of the protein without considering the purification tag, and translational and rotational artifacts were removed by fitting the coordinates to the position of the α-carbons (Cα). The probability of an amino acid being involved in a secondary structure (i.e., α-helix, β-sheet, turn, or coil) was assessed using the MDTraj Python library, which utilized the Dictionary of Protein Secondary Structure (DSSP) program^27^ as the backend. The secondary structure probability for each amino acid was calculated by dividing the total number of occurrences of a specific secondary structure by the total number of frames in the concatenated trajectories. The probability of a specific internal interaction between residue 1611 and the rest of the protein (i.e., hydrogen bonds, hydrophobic interactions, salt bridges, metal complexes, etc.) was evaluated using the PLIP Python library^28^ for each frame and then averaging the number of occurrences over the total number of frames. Interactions with a probability higher than 30% were selected, according to previous studies.^9,29^

The T-REMD simulations were characterized employing two collective variables: the beta root mean squared deviation (βRMDS) and a distance (d) (Figure S2).

The parallel and antiparallel βRMSD measure was employed to characterize the secondary structure content of the amino acid in the region 1532–1542. The βRMSD is a collective variable that provides a measure of structural similarity, emphasizing the local secondary structure arrangements by comparing pairs of backbone segments to that of an ideal β-sheet.^30^ The βRMSD was evaluated as the sum of the parallel and antiparallel content of βRMSD. Moreover, the Cartesian distance, d, between the Cα of residue 1611 and the center of mass of Cα in the region 1545–1550 was evaluated. For the analysis of the REMD trajectory, the first 50 ns of simulations were discarded. Then, the trajectory was analyzed through the block averaging method. The last 150 ns was divided into 10 ns windows. From each window, the bivariate histogram of the probability distribution, H­(V), was estimated in the space of V­(βRMSD,d). Then, an average of the histogram of the probability distribution, *H̅*, was estimated.

2 $${\mathrm{H̅}}_{i}=\frac{1}{N}\overset{N}{\underset{j=0}{\sum }}H_{i,j}$$

Finally, the free energy surface F along the space of βRMSD and distance were estimated, together with the associated error, through Boltzmann inversion according to ref. 31.

3 $$F_{i}=-k_{b}T\left[\ln\left({\mathrm{H̅}}_{i})-\ln(\max\left({\mathrm{H̅}}_{i}\right)\right)\right]$$

where *k*_b_ is the Boltzmann constant, *T* is the temperature, p­(H) is the probability, and *p*_max_ is the maximum value of the average probability distribution, to ensure that the minimum of *F* is equal to 0 kJ/mol.

### 2.9 Visualization, Plot, and Analysis Packages

Analyses were performed employing GROMACS tools,^32^ PLUMED,^33^ specific Python packages, i.e., MDAnalysis^34^ and MDTraj,^35^ and in-house scripts. Analysis plots were obtained with the Matplotlib library,^36^ while molecular system rendering and visual inspections were conducted using the Visual Molecular Dynamics (VMD) software^37^ and ChimeraX.^38^

## 3 Results

### 3.1 Biochemical Characterization of VPS9 WT and R1611W Mutant

After extraction and purification, SDS-PAGE was employed to evaluate the sample quality (Figure S3). Results showed that the final samples of both domains are >95% pure. We investigated the oligomerization state of VPS9^WT^ and the VPS9^R1611W^ mutant using size exclusion chromatography (Figure 1A). The wild-type domain elutes as a single but rather wide peak with an intensity maximum of 13.4 mL, which corresponds to a population endowed with an apparent molecular weight (MW_app_) of ∼64 kDa. The elution profile suggests that the VPS9^WT^ domain (MW_VPS9 monomer_ = 16.6 kDa) exists mainly as a VPS9 tetrameric assembly under our experimental conditions. On the contrary, the VPS9^R1611W^ mutant also elutes as a rather wide peak that, however, displays an absorbance maximum at 14.1 mL and three “shoulders,” at 13.4, 12.4, and 11.2 mL, respectively, indicating that the R1611W mutation affects the VPS9 homo-oligomerization state. According to the column calibration (Figure S4), the main population (14.1 mL peak) is endowed with a MWapp of ∼48 kDa. Such an apparent molecular weight could be compatible with a trimeric state of VPS9 but also with the existence of a rapid dimer-tetramer equilibrium not resolved by the column. The detected “shoulders” at 13.4 and 12.4, corresponding respectively to apparent molecular weights of ∼64 and ∼112 kDa, are compatible with the presence of minor but detectable tetrameric and hexameric VPS9 populations in solution.

**Figure 1:**
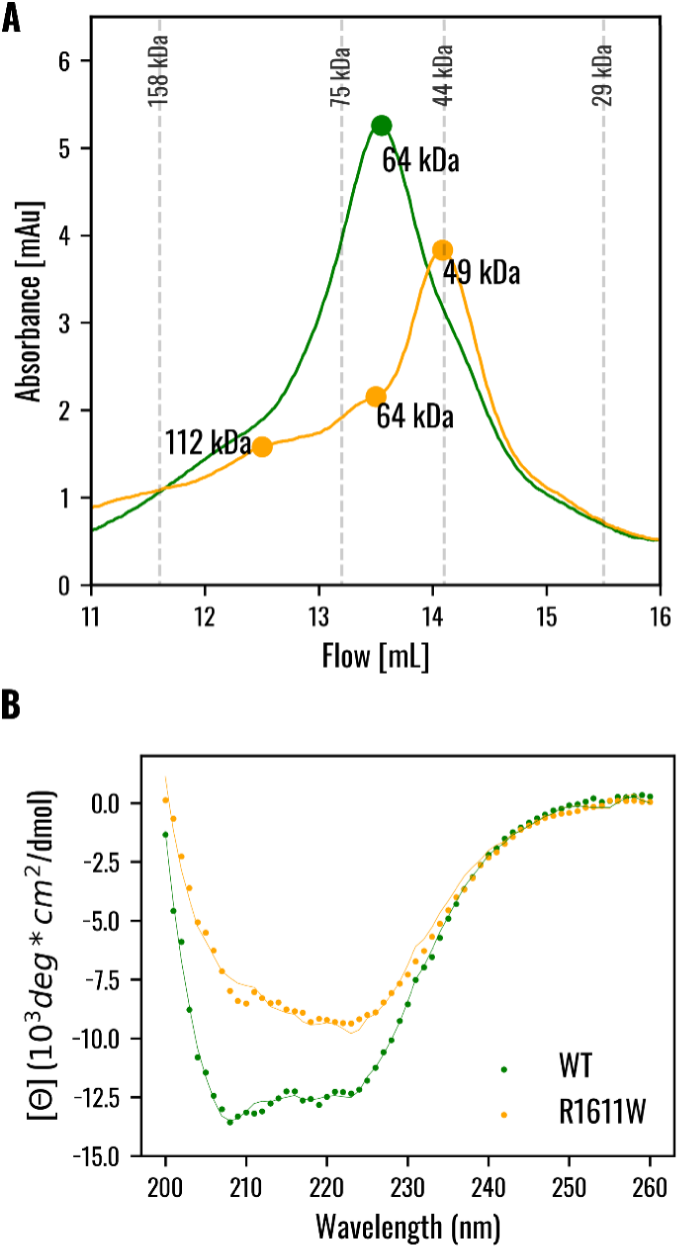
Biochemical characterization of the wild-type and mutant VPS9 by size exclusion chromatography and circular dichroism. (**A**) Size exclusion chromatography profiles correspond to 310 μg of VPS9^WT^ and 270 μg of VPS9^R1611W^ mutant eluted at a 0.5 mL/min flow rate in 50 mM potassium phosphate, pH 8.0, and 150 mM NaCl. Protein separation was performed at room temperature using a Superdex 200 Increase 10/300 GL column (Cytiva). The gel filtration column was calibrated with protein standards (Figure S4), whose elution volumes are reported as dashed lines with the corresponding molecular weight. (**B**) Far-UV circular dichroism postprocessed with SESCA: the solid line represents the calculated CD spectrum, while the dashed line represents the experimental CD spectrum. Results reporting VPS9^WT^ are shown in green, while VPS9^R1611W^ mutant results are shown in orange.

Circular dichroism has been employed to assess the refolding process and to give structural insights into the protein secondary structure composition. The DS6–1 basis set of the SESCA program was employed to deconvolve the secondary structure content as it showed the lowest RMSD between the calculated and the experimental CD spectrum (VPS9^WT^: 0.207 kMRE; VPS9^R1611W^: 0.338 kMRE).

Both CD spectra showed relative minima in the wavelength at ∼208 and ∼222 nm, typical hallmarks of an α-helix folding content (Figure 1B). However, the two spectra showed a different shape, which could be derived from a different folding of the structures. This is also evident from the secondary structure content derived from the SESCA deconvolution process (Figure 1B), which shows differences in terms of secondary structure composition. Both VPS9^WT^ and VPS9^R1611W^ seem to have a prevalence of α-helix (VPS9^WT^: 41.1%; VPS9^R1611W^: 30.2%) and unstructured regions (VPS9^WT^: 48.5%; VPS9^R1611W^: 48.6%) and a minor content of β-strand (VPS9^WT^: 10.6%; VPS9^R1611W^: 21.2%).

### 3.2 Molecular Model of VPS9

The molecular model of VPS9 was obtained, as described in the Materials and Methods section. All models showed negative Z-scores (VPS9^WT^: −5.65; VPS9^R1611W^: −5.04) in the region of experimentally resolved structures (Figure S5). The Ramachandran plot showed a good stereochemical quality of the models, with no amino acids in the disallowed region for VPS9^WT^ and seven residues (<10%) in the disallowed region for VPS9^R1611W^ (Figure S6). Both models showed similar secondary and tertiary structure predictions. In greater detail, VPS9^WT^ displays a globular shape formed by six helices (Figure 2A,B), similar to other VPS9 domain-containing proteins such as Rabex-5 ^39^ and VPS9a^40^ (more details are provided in the Supporting Information). As previously suggested by sequence analysis in the literature,^39^ the structure lacks an additional C-terminal α-helix, unlike Rabex-5 or VPS9a. However, unlike previously reported VPS9 structures, VPS9^WT^ showed a β-strand motif (aa 1533–1544 only beta sheet, or 1530–1553 full between the two helices) between α1 and α2 helices (Figure S7). Interestingly, the mutated alsin VPS9^R1611W^ showed a similar secondary structure distribution except for the β-strand motif, which was predicted as a disordered loop in the VPS9^R1611W^ structure (Figure 2A,B). Moreover, for both VPS9^WT^ and VPS9^R1611W^, the purification tag was predicted to be disordered and not participate in the arrangement of the protein. The averaged pLDDT was 83.28 for the whole VPS9^WT^ predicted structure and increased to 88.54 when the purification tag was excluded. On the contrary, for VPS9^R1611W^, the average pLDDT was lower, considering both the whole structure (77.67) and the VPS9 region without the purification tag (83.53). This difference was mainly due to a lower local pLDDT in both the purification tag/α1 region and the β1 region (Figure S7). Notably, the predicted pLDDT values for residue 1611 show no substantial difference between wild-type VPS9 (94.80) and the R1611W mutant (92.87), suggesting that this substitution does not significantly affect the local structural confidence of that residue (Figure S8). To assess the effect of the presence of the purification tag on the predicted structure of VPS9^WT^, we compared the predicted structure with that of the whole alsin retrieved from AlphaFolddb, as reported in the Materials and Methods section. The comparison showed a low difference in terms of RMSD (1.13 Å), with major differences in the terminal regions from 1654 to 1657 (Figure S9). The residue 1611 is predicted to be at one end of the α4 helix for both VPS9^WT^ and VPS9^R1611W^ (Figure S10). The WT R1611 was predicted to be almost buried in the helix bundle, with an accessible surface area (ASA) lower than 1% (ASA = 1.7 Å^2^). On the contrary, in the mutant, where the arginine was mutated to tryptophan, around 17% of the surface of the residue was predicted to be exposed to the solvent (ASA = 54.9 Å^2^).

**Figure 2:**
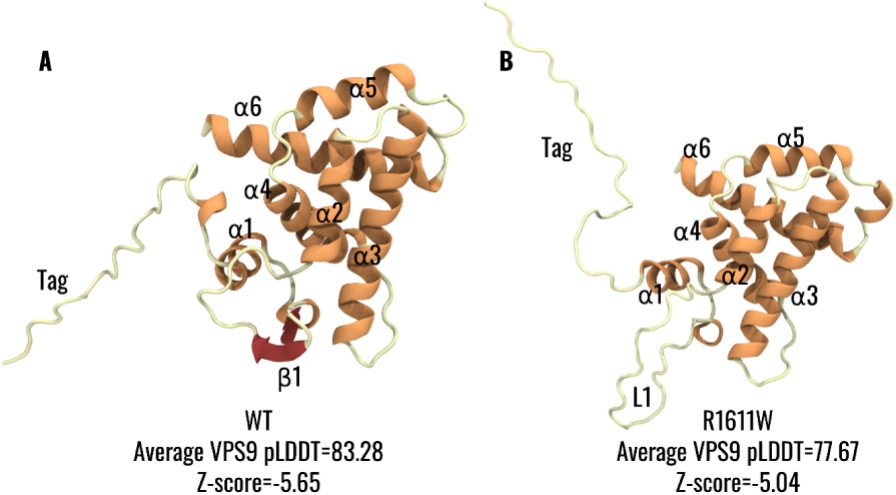
VPS9 molecular models representative of (**A**) VPS9^WT^ and (**B**) VPS9^R1611W^; both models were rendered in cartoon representation and colored according to the secondary structure (helices in orange, sheets in red, and coils in light yellow).

### 3.3 Structural Characterization of the Alsin VPS9 Domain

We then performed a molecular dynamics simulation of both models to investigate the behavior of the VPS9 monomer at the molecular scale and the effect of the mutation on the local interactions of VPS9 by performing an analysis of the nonbonded interactions that residue 1611 creates with the rest of the protein, both in the WT and mutated proteins.

Equilibrium of the simulation was assessed through RMSD analysis, with all of the simulations reaching equilibrium after 1.5 μs (Figure S11). The analyses were conducted on the last 500 ns of each replica, i.e., the equilibrium portions, sampled at 0.1 ns.

Secondary structure content extracted as an average from MD simulations slightly differed compared to that determined by circular dichroism (Figure 3A). Indeed, both VPS9^WT^ and VPS9^R1611W^ seemed to have a higher α-helix content (VPS9^WT^: 50.5 ± 2.1%; VPS9^R1611W^: 51.5 ± 2.26%), while unstructured regions seemed to have a similar distribution (VPS9^WT^: 46.1 ± 3.2%; VPS9^R1611W^: 48.1 ± 2.5%), although a minor content of β-strand (VPS9^WT^: 3.4 ± 2.4%; VPS9^R1611W^: 0.4 ± 0.7%) is predicted from both models VPS9^R1611W^ and VPS9^WT^. Moreover, the content of β-strand is higher in VPS9^WT^ compared to VPS9^R1611W^, showing an opposite trend compared to circular dichroism. More specifically, the difference in β-strand content is due to the region of β1, which is conserved as a sheet in VPS9^WT^ throughout the simulation time (Figure 3B), while in the same region, VPS9^R1611W^ showed a coil structure that does not fold into a β-strand (Figure 3B).

**Figure 3:**
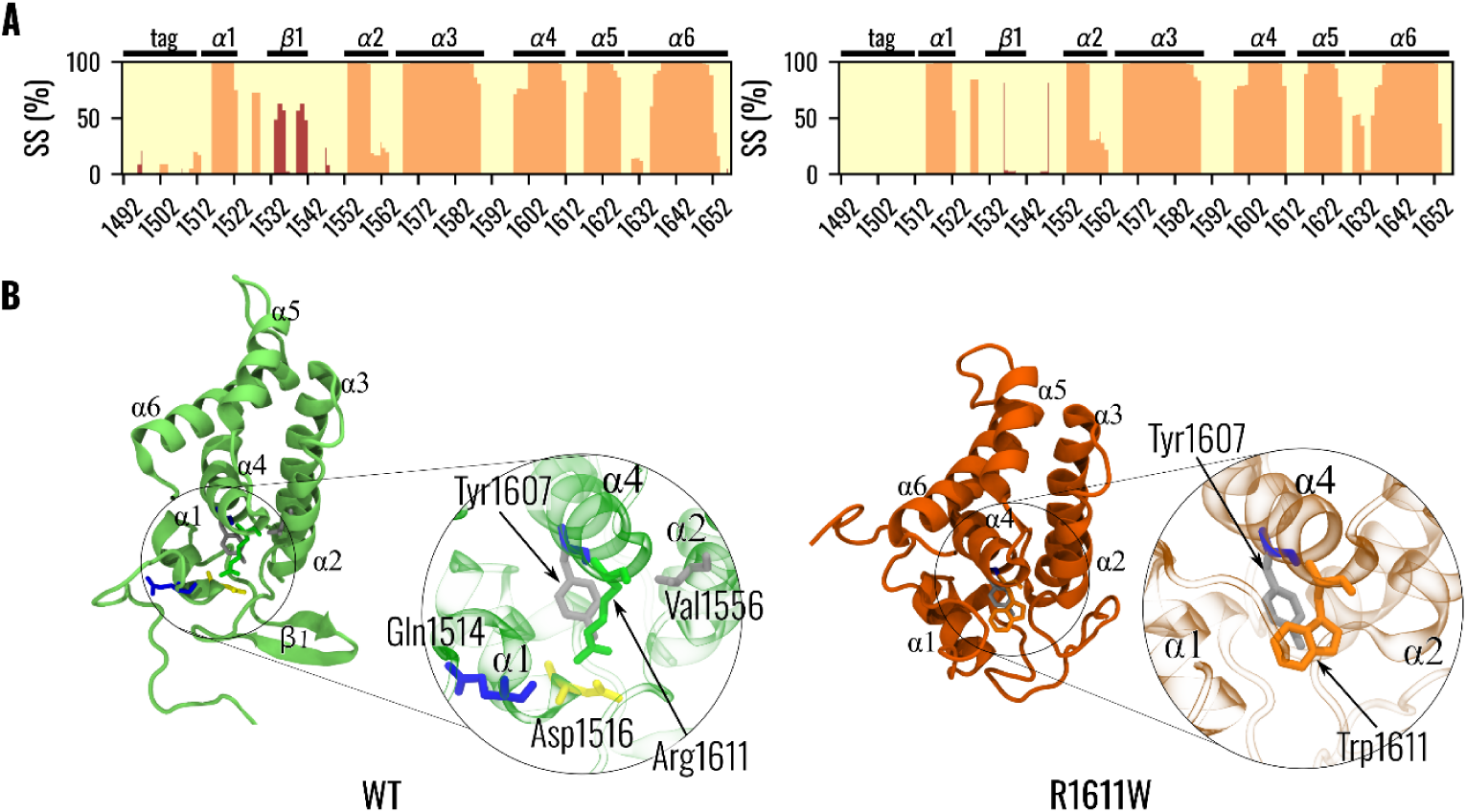
Dynamical characterization of the VPS9 models: green VPS9^WT,^ orange VPS9^R1611W^. (**A**) Secondary structure probability for each residue: secondary structure elements for VPS9^WT^ (top panel) VPS9^R1611W^ (bottom panel) are colored according to the following scheme: helices in orange, sheets in red, and coils in light yellow. (**B**) Dynamical characterization of amino acid 1611 interactions: VPS9^WT^ and VPS9^R1611W^ mutant structures and the corresponding residue 1611 are colored in green and orange, respectively. The VPS9 structure is shown in a cartoon representation, while residues are shown as sticks. Colors of interacting residues represent their interaction according to the following scheme: salt bridges in yellow, hydrogen bonds in blue, and hydrophobic interactions in gray.

We subsequently analyzed the interaction between residue 1611 and the rest of the protein to assess the local effect of the mutation. An initial analysis of interactions was performed on the VPS9 structure predicted by AlphaFold. Considering R1611 in VPS^WT^ the amino acid backbone is predicted to be involved in a hydrogen bond with tyrosine 1607, while the guanidyl side chain of the amino acid is involved in a hydrogen network with two aspartic acids belonging to α1 (D1516) and to the coil region between α1 and α2 (D1549), respectively (Figure S10A,C). On the contrary, in the mutant, the backbone retains its interaction with Y1607 (Figure S10B,D); however, the mutation-induced substitution alters the side chain from polar to aromatic, leading to the disruption of the hydrogen bonding network. We can surmise that upon mutation into a tryptophan, the electrostatic interactions between R1611 and the mixed β-sheet coil in between the α1 and α2 helices (and more precisely D1516 and D1549) are abolished, and the bulky apolar tryptophan side chain in the mutant VPS9 may create steric hindrance, which may then repulse and destabilize this portion (Figure S12).

A second analysis of interaction probability was performed on the molecular dynamics trajectory of the two structures. The interaction probability for R1611 is detailed in Table 1. In VPS9^WT^, R1611 was consistently involved in a hydrogen bond with Y1607, along with a hydrophobic interaction. Moreover, R1611 formed a salt bridge with D1516 and Q1514, located on the α1-helix, and made less probable hydrophobic contact with residue V1556, bridging together the α1 and α2 helices (Figure 3B). However, the interaction with residue D1549 was not maintained. On the contrary, it is important to point out that D1549 shows a high probability of forming a hydrogen bond with D1512. However, the interaction of R1611 with the D1549 residue was lost during dynamics. It is also worth mentioning that D1549 shows a high probability (∼60%) of forming hydrogen bonds with D1516, remaining in the vicinity of R1611 and bridging α1 and β1 together.Table 1

**Table 1:** Most Probable Interactions with the amino acid R1611

Type	Residue	Probability (%)
Hydrogen bond	Y1607	99
Salt bridge	D1516	80
Hydrophobic	Y1607	73
Hydrogen bond	Q1514	56
Hydrophobic	V1556	73

Notably, the mutated W1611 residue showed fewer overall noncovalent interactions, as reported in Table 2. While W1611 hydrogen bonding to Y1607 was retained for the mutated VPS9^R1611W^, together with a hydrophobic interaction, no other interactions were observed (Figure 3B). Most notably, the interaction between residues D1516 and D1549 was lost.

**Table 2:** Most Probable Interaction with the amino acid W1611

Type	Residue	Probability (%)
Hydrogen bond	Y1607	99
Hydrophobic	Y1607	46

Results from protein structure prediction and classical MD suggest a role for the mutation in destabilizing the β1 region. Therefore, we carried out T-REMD simulations to explore the folding of the β1 region. Two collective variables were employed to characterize the energy profile: the βRMSD monitors the content of β structure, and a distance, d, as described in the Materials and Methods section, evaluates the proximity of residue 1611 and the loop neighboring the β1 region. Figures S13–S16 provide more details on the exchange between replicas. The bivariate distribution shows a sudden change in the energy profile because of the mutation (Figure 4A; see also Figure S17 for the related error). Indeed, the energy profile showed three basins for VPS9^WT^: a region **A,** where the loop is at around 17.5 Å from residue 1611 and β1 is unstructured; and two regions, **B** and **C**, where the loop and residue 1611 are in close contact at around 12 Å and are characterized by unstructured and structured β1 behavior, respectively.

**Figure 4:**
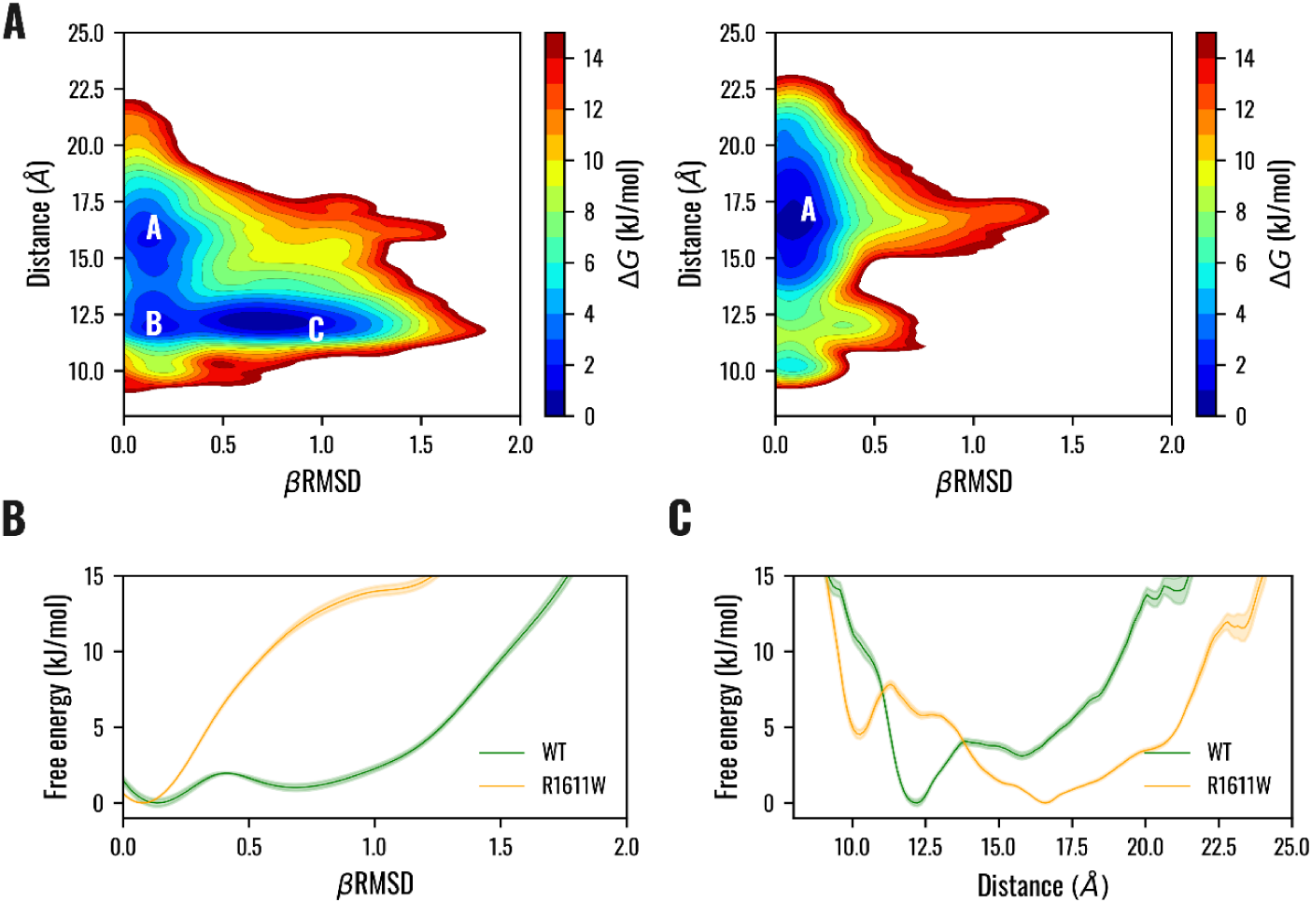
Free energy surface along the βRMSD of the region 1533–1543 and the distance d between the Cα of residue 1611 and the center of mass of the Cα of loop 1545–1550 for VPS9^WT^ and VPS9^R1611W^ mutant. (**A**) Free energy of VPS9^WT^ (left) and VPS9^R1611W^ mutant (right) as a function of the βRMSD and the distance d are reported in kJ/mol. (**B**) The 1D projection of the energy profile along the βRMSD (solid line) and the relative error in the estimation (shaded). (**C**) The 1D projection of the energy profile along the distance (solid line) and the relative error in the estimation (shaded).

Moreover, the energy profile of the VPS9^R1611W^ mutant is characterized by a deep well of potential, **A**, where the loop fluctuates between 15 and 20 Å from residue 1611, and the β1 region is unstructured. The energy profile along the βRMSD of the β1 region for the VPS9^WT^ mutant is characterized by two minima between 0 and 1, divided by an energy barrier of around 2 kJ/mol, suggesting a dynamic equilibrium between the folded and unfolded states of the β1 region Figure 4B. On the contrary, the VPS9^R1611W^ profile is characterized by a single minimum at around 0 βRMSD, with no other minima, suggesting that the mutation alters the equilibrium, driving the β1 region to an unstructured behavior (Figure 4B).

## 4 Discussion

Mutations in the *ALS2* gene, including the R1611W variant in the VPS9 domain, are associated with rare yet severe neurodegenerative conditions such as IAHSP. Although relevant at the clinical level, the structural mechanisms by which these mutations impair alsin function remain largely unclear. This study combines *in vitro* and *in silico* methods to elucidate how local conformational changes from pathogenic mutations can hinder the folding and oligomeric assembly of the VPS9 domain, possibly contributing to disease onset.^1^

Our results first highlight a clear impact of the R1611W mutation on the quaternary structure of the VPS9 domain. The VPS9^WT^ protein was shown to form stable tetrameric assemblies (Figure 1A), a behavior consistent with homologous VPS9 domains in other proteins, such as Rin2, where oligomerization has been similarly attributed to the VPS9 domain.^41^ In contrast, the mutant VPS9^R1611W^ displayed oligomeric behavior consistent with either trimeric assemblies, a more compact tetramer, or a dynamic equilibrium between dimeric and tetrameric forms (Figure 1A). Such deviations from the wild-type oligomeric state reflect a destabilization of the native quaternary structure, consistent with previous observations on the full-length protein,^11^ and suggest that the R1611W residue plays a structural role in promoting a specific oligomerization pattern, potentially critical for functional alsin activity. It is important to underline, however, that our results pertain only to the VPS9 domain and cannot provide direct insights into the oligomerization behavior of full-length alsin, which lies beyond the scope of this work. Instead, this study focuses on local structural effects within the VPS9 domain that could influence its molecular behavior.

To investigate whether alterations in secondary structure occur alongside changes in quaternary organization, we examined the folding signatures of the two proteins using circular dichroism spectroscopy. Far-UV CD spectra suggest that the VPS9 WT secondary structure has ∼40% α-helices, which aligns with VPS9 domains from other proteins^39,42^ (Figure 1B). One example is Rabex5, another GEF exchange factor, whose three-dimensional crystal structure shows a high α-helical content.^39^ This observation reinforces that the α-helical content is a conserved structural feature among VPS9 domains with functional GEF activity.

Surprisingly, the R1611W mutant displays a different content of secondary structures, with a reduced α-helix content in favor of an increased β-sheet content (Figure 1B). Such a shift in the folding pattern is indicative of mutation-induced destabilization and misfolding, extending beyond oligomerization defects. Compared to Rabex5, which exhibits a stable six-helix bundle architecture in the VPS9 region, the altered folding observed in the mutant may reflect a loss of structural motifs essential for functional Rab binding and exchange activity. These findings suggest that the R1611W mutation might perturb the structural integrity of the domain, potentially compromising the VPS9 machinery.

Atomistic modeling provided the structural framework to interpret the CD data and assess whether the observed changes in secondary structure reflect specific alterations in the VPS9 fold. In this context, our structural modeling of the VPS9 domain revealed an overall architecture predominantly composed of α-helices (Figure 2A), consistent with known VPS9 structures.^39,42^ However, the structural model of VPS9^WT^ was characterized by a region showing a β-hairpin structure between the α1 and α2 helices (Figure 2A), notably in spatial proximity to residue 1611, which is not observed in other crystallographic VPS9 domains, solely composed of a six α-helix bundle (α1–6).^39,42^ This β-hairpin motif, although absent in other VPS9 structures, may represent a unique and functionally relevant feature of the alsin VPS9 architecture. In contrast, the VPS9 mutated model lacked this β-hairpin motif, displaying instead a disordered coil in the same region, suggesting a local destabilization caused by the mutation (Figure 2B).

Its absence in the mutant and its closeness to the mutated residue strongly indicate that R1611 plays a causative role in stabilizing this structural element. This local unfolding may also anticipate a shift toward increased disorder, which is further supported by the dynamic and confidence-based analyses described hereafter.

Consistent with the structural rearrangement observed in the mutant model, the pLDDT value of the β-hairpin region assumes values of around 70 for the WT and below 40 for the R1611W mutant (Figure S8A). This marked drop in local confidence suggests that the R1611W mutation inhibits the formation of the β-hairpin and increases the propensity for local flexibility or disorder. More specifically, such a low pLDDT score may reflect both reduced prediction confidence and the presence of intrinsically disordered regions within the protein.^20,43^ In line with this observation, the β-hairpin region may act as a transiently folded element whose stability is finely tuned by local interactions involving R1611. Disordered regions in proteins are known to be key actors in the dynamic function of eukaryotic proteins.^44,45^ In this context, such local disorder may reflect a regulatory structural feature whose disruption could interfere with proper VPS9-mediated functions.

These observations raised the question of whether R1611 contributes directly to maintaining the β-hairpin architecture through specific intramolecular interactions.

Our simulations suggested that in VPS9^WT^, R1611, together with D1516 and D1549, creates a network of interactions (Figure 3B) that stabilizes the formation of the β-strand region. Upon substitution with tryptophan, the loss of these electrostatic interactions leads to a disruption of the packing between α4, α1, and the β-strand, thereby destabilizing the local fold.

Interestingly, we also observed that in one of the classical MD replicas, the β1 region of the VPS9^WT^ transitioned to an unstructured coil. In contrast, the mutant failed to show any reversal from the coil to β-structure, suggesting a reduced capacity to refold. However, this type of transition may occur over longer time scales, and simulations extending to hundreds of microseconds may be necessary to fully capture the folding equilibrium. The conformational plasticity observed in the β1 region suggested a potential regulatory role, prompting further analysis of how residue 1611 modulates this behavior. REMD simulations revealed that in the WT condition, the β1 region explores both folded and unfolded conformations, suggesting an intrinsic dynamic flexibility. The role of residue 1611 appears crucial in this equilibrium as it creates a network of electrostatic interactions between its positively charged side chain and negatively charged residues D1516 in helix α1 and D1549 in the loop region neighboring the β1. Moreover, REMD results suggest a dynamical behavior of this loop, which can switch between folded and unfolded conformations separated by energy differences on the scale of thermal fluctuations (Figure 4). In the R1611W mutant, this equilibrium is significantly perturbed: the apolar and bulky side chain of tryptophan fails to sustain the electrostatic network. Consequently, it reduces the ability of the region to switch between distinct conformational states (Figure 4).

In summary, the conformational adaptability of the β1 region appears to rely on a stabilizing electrostatic network involving residue R1611. The disruption of this network in the R1611W mutant abolishes the region’s ability to reversibly sample folded and unfolded conformations, suggesting that local flexibility is lost.

This dynamic behavior may not be merely structural: in the broader context of VPS9 domains, conformational changes and intramolecular regulatory elements are known to regulate their GEF function, which catalyzes nucleotide exchange on Rab GTPases—for example, through autoinhibition mechanisms or activation upon ubiquitin binding.^42,46^ Although such mechanisms have not been fully characterized in alsin, our findings raise the possibility that the β1 region—and its ability to fluctuate between conformational states—could play a regulatory role in the activation of downstream Rab pathways.

The loss of this equilibrium in the mutant may thus represent a mechanistic link between the local structural defect and the impaired function observed in the disease-related forms of alsin. In conclusion, this work provides a structural characterization of the human alsin VPS9 domain, highlighting its conformational features associated with monomeric and oligomeric forms. Importantly, computational results enable speculation about the molecular consequences of the pathological R1611W mutation. We detailed that the mutation is responsible for disrupting a local electrostatic network that stabilizes the β1 region, leading to misfolding, reduced conformational plasticity, and potentially altered oligomerization. R1611W-induced conformational alterations are also highlighted by experimental observations, although with some contextual differences from computational investigations. Whereas circular dichroism indicates an increased β-sheet content in the mutant, *in silico* analyses predict local unfolding and enhanced disorder around the mutation site. This apparent divergence likely reflects the different molecular contexts probed: circular dichroism captures the global secondary structure of oligomeric species present in solution and identified by size exclusion chromatography, whereas simulations focus on the pure monomeric forms, which are devoid of interactions with other VPS9 monomers. It is plausible that β-sheet elements emerge upon self-oligomerization.

Altogether, the findings indicate that the R1611W mutation undermines the structural integrity and dynamic adaptability of the VPS9 domain, hindering the proper assembly and function—thereby establishing a mechanistic connection to its role in IAHSP.

## Supplementary Material



## Notes

Structural, biochemical, and simulation analyses of the alsin VPS9 domain and its R1611W variant; a schematic overview of alsin’s domain organization and a full-length AlphaFold structural prediction; collective variables for VPS9 dynamics defined through βRMSD across residues 1532–1542 and the distance between residue 1611 and residues 1545–1550; biochemical characterization that includes SDS-PAGE analyses showing VPS9 WT in inclusion bodies and the purification of both WT and R1611W proteins, complemented by size exclusion chromatography calibration; model quality assessments comprising ProSa Z-score plots, Ramachandran diagrams, and per-residue pLDDT profiles with color-coded structural renderings, as well as comparative structural alignments with *Arabidopsis* VPS9a and Rabex5 homologs; further structural insights including the β-strand region highlighting the R1611–D1549 electrostatic interaction in WT and its disruption in the mutant, along with residue-level comparisons of R1611 and W1611 environments; molecular dynamics analyses that feature RMSD trajectories of VPS9 WT and R1611W, structural comparisons with full-length alsin models, and replica exchange molecular dynamics simulations, including the full temperature ladder, exchange probabilities, and potential energy distributions and error estimations of free energy profiles for both WT and mutant VPS9 models (PDF)
