# Supplementary material for: Molecular Insights into IAHSP: Influence of the R1611W Mutation on the VPS9 Domain of Alsin
Source: ACS Omega. 2025 Nov 5;10(45):54138–47. doi: 10.1021/acsomega.5c05926 (PMC12631311; doi:10.1021/acsomega.5c05926)
Supplement: Supplementary file 1 [file ao5c05926_si_001.pdf]

Supporting Information

Molecular insights into IAHSP: influence of the R1611W mutation on the VPS9 domain of alsin

Authors: Marcello Miceli[1,2], Cécile Exertier[3], Elena Gugole[3,4], Beatrice Vallone[4], Marco Agostino Deriu[1*]

[1] Polito[BIO]Med Lab, Department of Mechanical and Aerospace Engineering, Politecnico di Torino, Italy

[2]Department of Life Sciences, Università Degli Studi di Modena e Reggio Emilia, Via Campi 103, Modena, 41125, Italy

[3]Institute of Molecular Biology and Pathology, Italian National Research Council (IBPM-CNR), c/o Department Biochemical Sciences, Sapienza University of Rome, Ed. CU027, P.le A.Moro 5, 00185 Rome, Italy;

[4]Department of Biochemical Sciences "ALESSANDRO ROSSI FANELLI", *Università La Sapienza*, Italy

* Corresponding author: marco.deriu@polito.it

1.1 Alsin domain and 3D structure

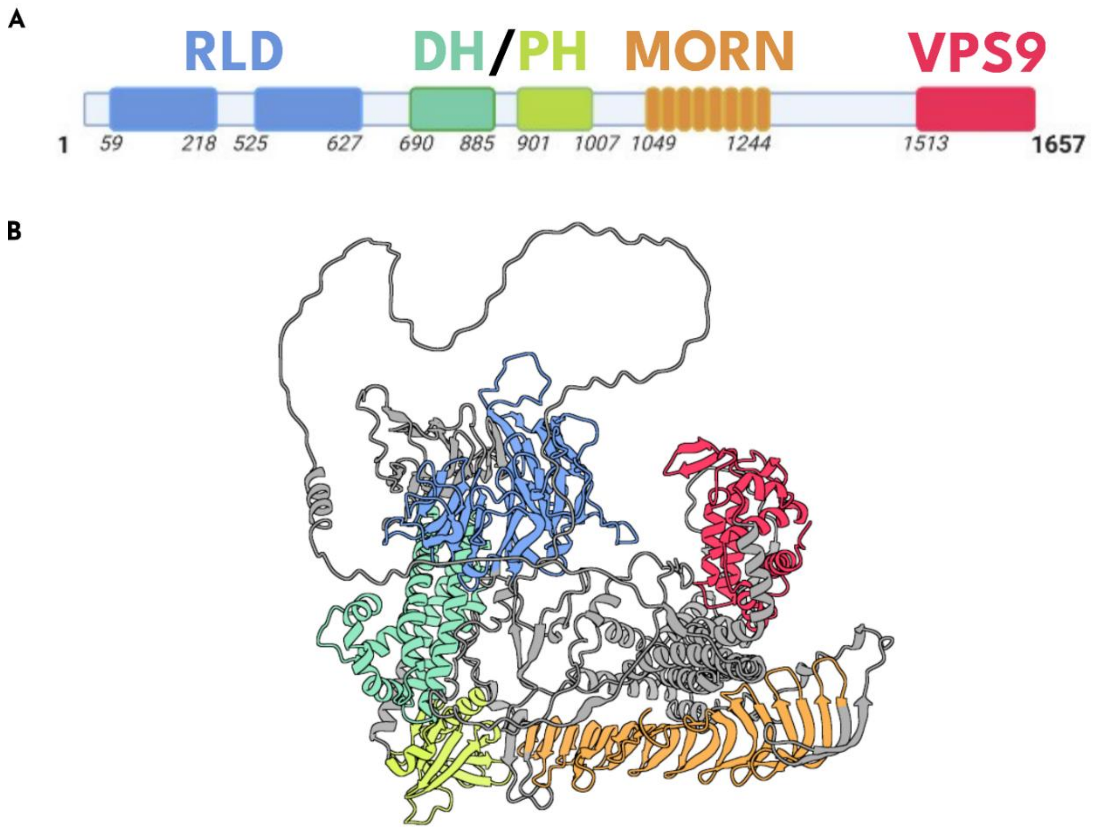

__Figure S1. Overview of alsin's domain organization and structural features. (A)__ Amino acid sequence and schematic representation of Alsin, with structured domains identified by homology modelling. Each domain is shown in a different color, and the positions of the first and last residues are indicated. __(B)__ Cartoon representation of the full-length Alsin protein structure retrieved from AlphafoldDB (UniProt ID: Q96Q42), with structured domains colored according to the schematic in panel A.

1.2 Collective variables to explore VPS9 dynamics

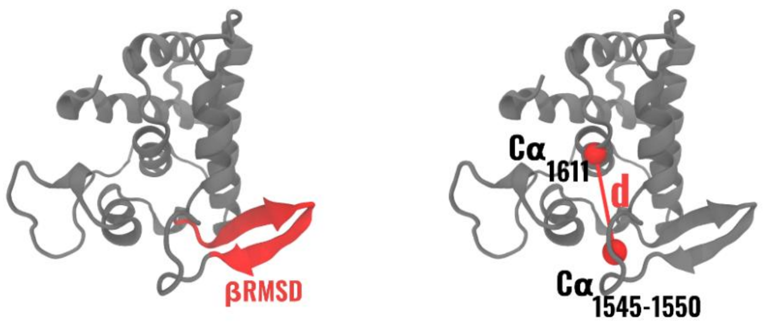

__Figure S2. Collective variable representation.__ On the left the region 1532-1542 for the calculation of the βRMSD collective variable, on the right the depiction of the distance calculated between the centre of mass of the Cα 1611 and 1545 to 1550.

1.3 Protein samples Expression and purification

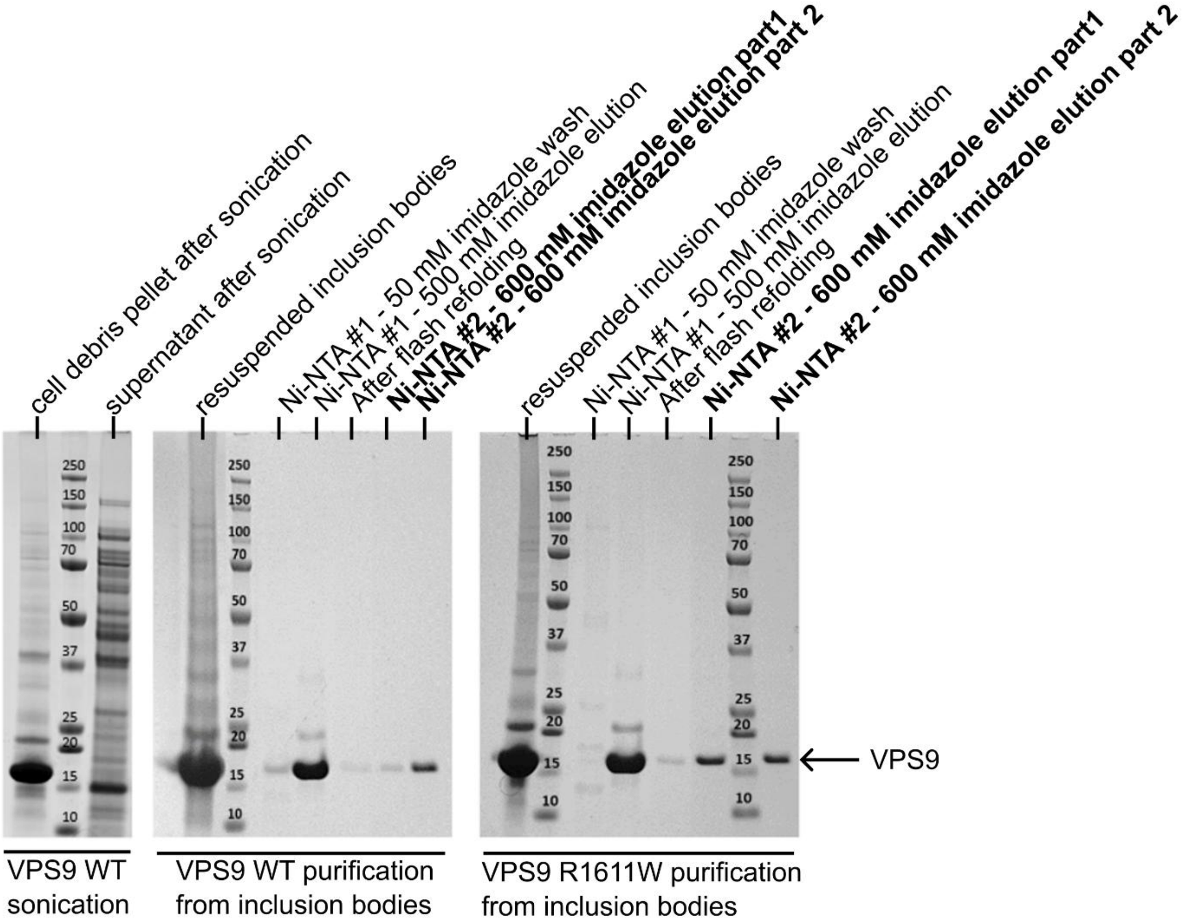

Figure S3. SDS-PAGE electrophoresis for the purification of the VPS9 WT and R1611W domains*. Samples were prepared with MPage LDS sample buffer 4X (Merck Millipore), and run onto a 12% mPAGE 12% Bis-Tris electrophoresis gel (Merck Millipore) in NuPage SDS running buffer (Invitrogen) using the Xcell SureLock mini-cell apparatus (Invitrogen) according to the manufacturer's instructions. After running, gels were stained with Coomassie brillant blue R 250 (Merck Millipore). The "VPS9 WT sonication" SDS-PAGE gel shows that VPS9 is present in inclusion bodies. The other two SDS-Page gels show the purification of both VPS9 WT and VPS9 R1611W domains from the inclusion bodies. For both domains, the resulting final samples display high purity. The bands corresponding to VPS9 are indicated with an arrow.*

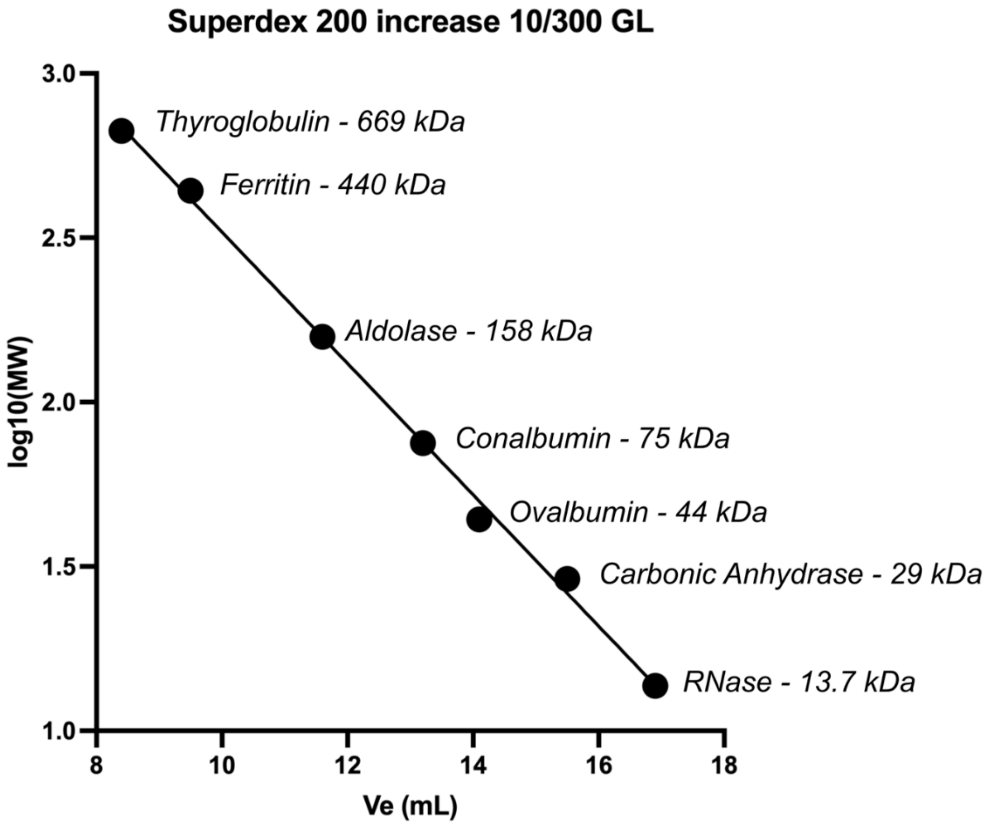

Figure S4. *Calibration of the Superdex 200 Increase 10/300 GL column.*

1.4 Analysis of alsin VPS9 models quality

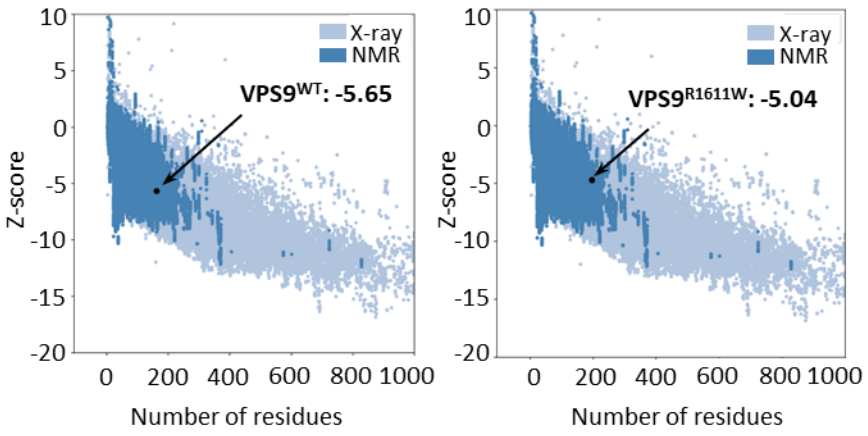

Figure S5. Visual representation of Z-scores against the number of residues*. Experimentally resolved structures from Protein Data Bank are represented as dark (NMR) or light (X-ray) blue dots, while VPS9 models as black dots. The Z-scores have been evaluated through the ProSa web server* [1].

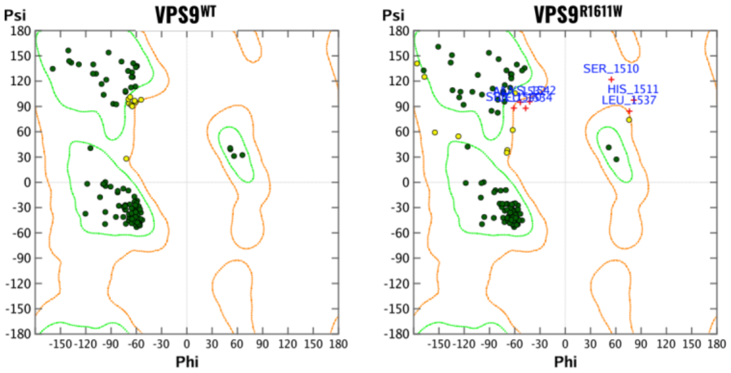

Figure S6. *Ramachandran plots for the two developed models.*

1.5 Sequence and structural comparison of Alsin VPS9 with experimentally resolved VPS9 homologues

A sequence and structural comparison was carried out to assess variability between the Alsin VPS9^{WT} predicted structure and other VPS9 homologues domains reported in the literature. Specifically, employing the MOE[2] alignment tool we compared Alsin VPS9^{WT} with the experimentally resolved structures of Arabidopsis thaliana VPS9a (AtVPS9a; PDB ID: 2EFD) and Rabex-5's VPS9 (Rabex5; PDB ID: 2OT3). The alsin VPS9 sequence, excluding the tag region, was aligned with those of AtVPS9a and Rabex5, and the structures were superimposed.

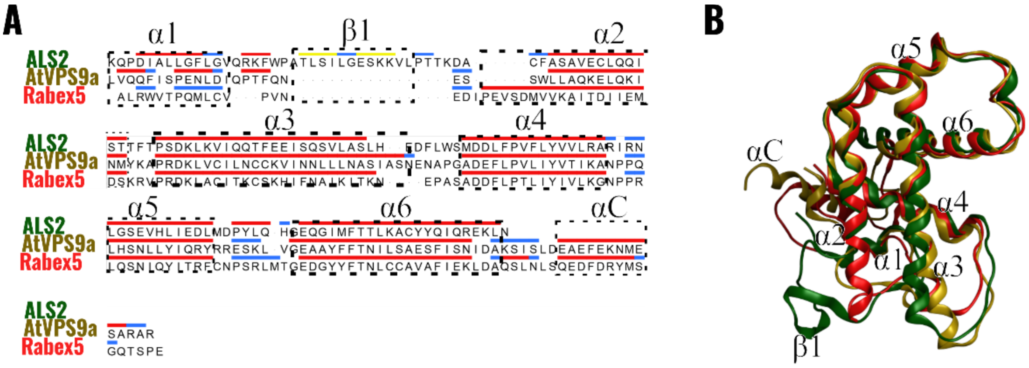

Figure S7. Comparison of the predicted structure of alsin's VPS9 domain with experimental VPS9 homologues. (A) *Sequence alignment of alsin VPS9 with AtVPS9a and Rabex5, with secondary structure elements indicated (α-helices in red, β-sheets in yellow, and turns in blue).* ***(B)*** *Structural alignment of the predicted VPS9 wild-type (VPS9^{WT}) with the experimental structures of AtVPS9a (PDB ID: 2EFD[3]) and Rabex5 (PDB ID: 2OT3[4]).*

1.6 Analysis of alsin VPS9 models prediction score and influence of the Tag region

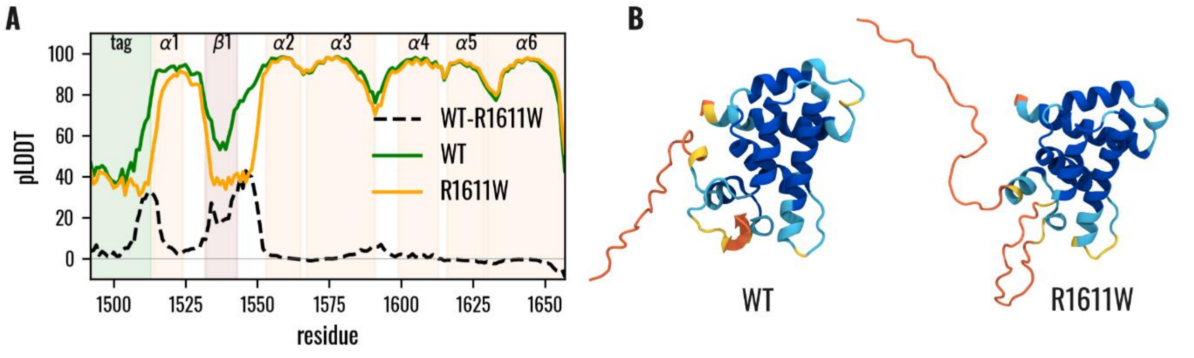

Figure S8. The predicted local distance difference test (pLDDT) for VPS9 WT and R1611W AlphaFold models ***(A)*** *pLDDT per residue for the VPS9 models, with the residue wise difference reported ad dashed black line,* ***(B)*** *a rendering of the models in new cartoon representations colored according to the valute of pLDDT (Blue pLDDT>90, Light blue 90>pLDDT>70, Yellow 70>pLDDT>50, orange pLDDT<50).*

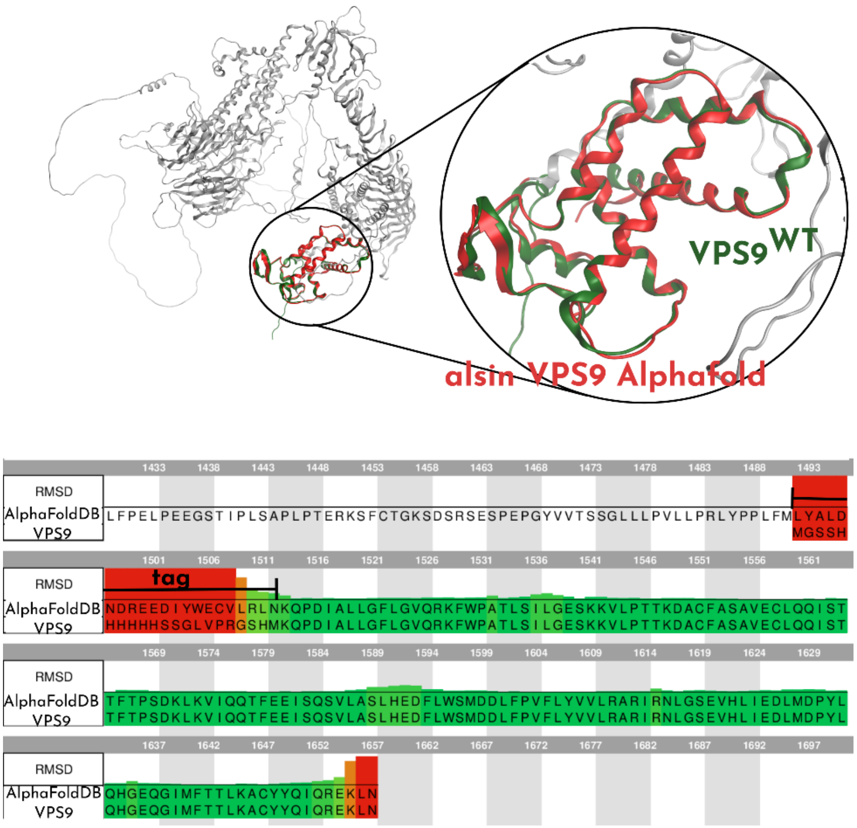

Figure S9. *Structural comparison between VPS9 predicted model (green) and the VPS9 in the alsin (red) model retrieved from AlphaFoldDB (gray) (UniProt ID: Q96Q42). The Cα per-residue Root mean squared distance is reported in the lower panel with green meaning values < 1 Å.*

1.7 Molecular dynamics simulation and interaction analysis

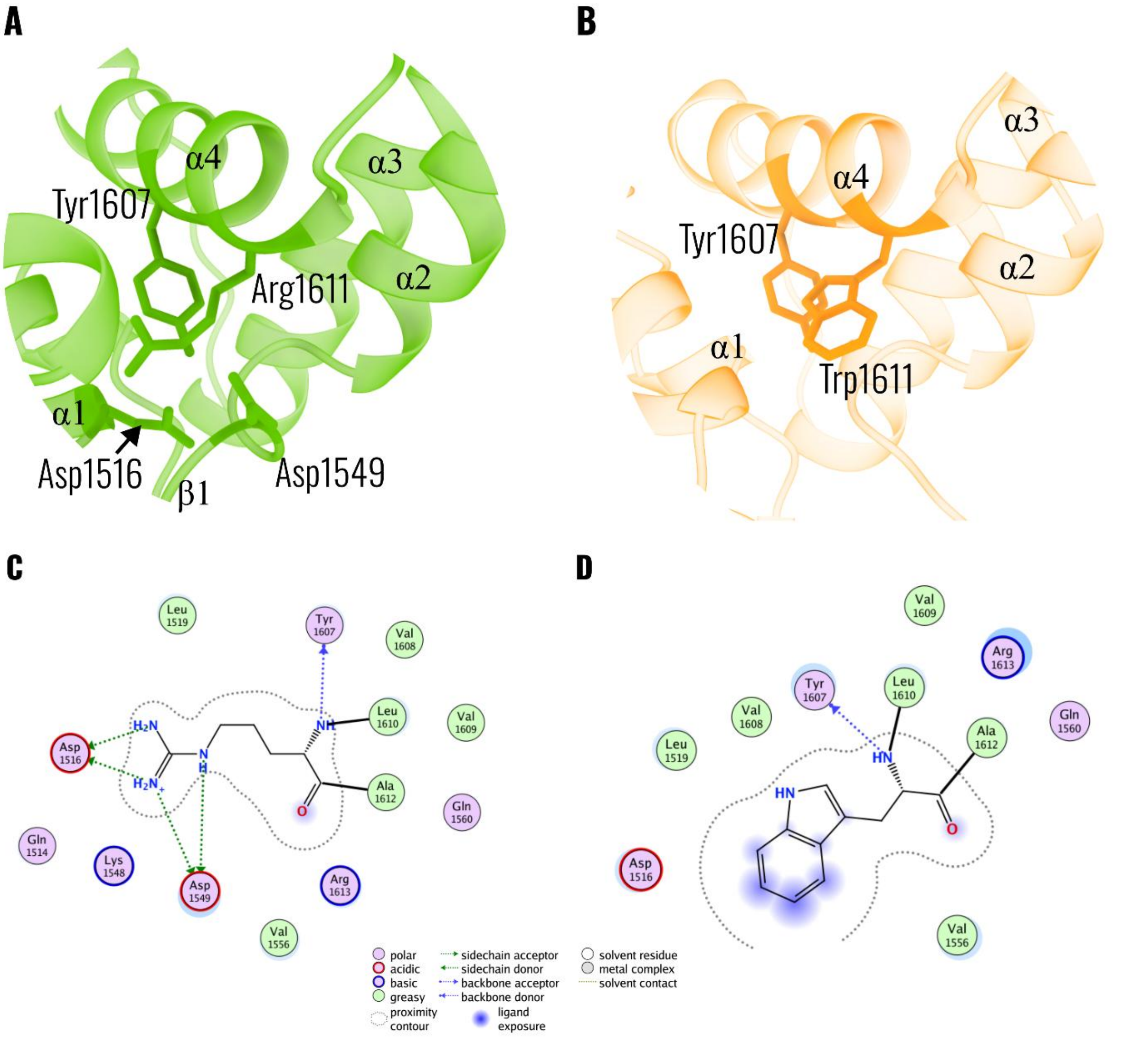

Figure S10. Interaction of the 1611 residue with the surrounding amino acids in the VPS9 AlphaFold predicted model (A) *Rendering of the residue R1611 and the interacting amino acids in the VPS9^{WT} model, the protein is represented as cartoon, interacting amino acids are rendered as licorice representation* ***(B)*** *Rendering of the residue W1611 and the interacting amino acids in the VPS9^{R1611W} model, the protein is represented as cartoon, interacting amino acids are rendered as licorice representation* ***(C)*** *Interactions of the wild-type R1611 residue with the surrounding amino acids of the VPS9^{WT} in the predicted model,* ***(D)*** *Interactions of the mutated W1611 residue with the surrounding amino acids of the VPS9^{R1611W} in the predicted model.*

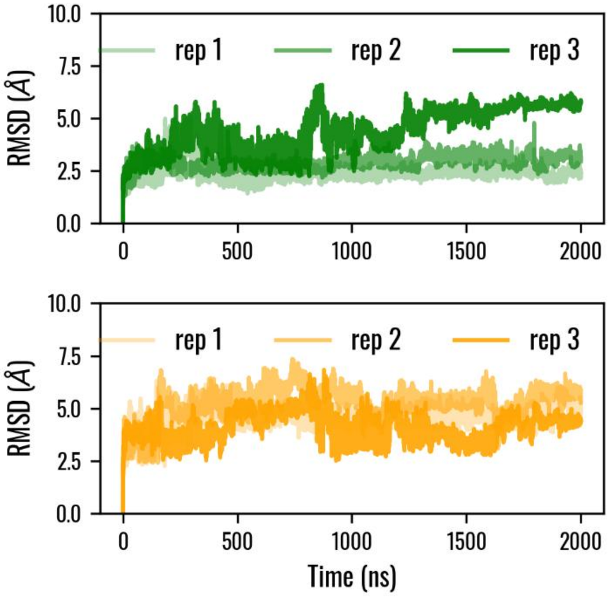

Figure S11. *RMSD of VPS9 Cα with respect to the initial configuration after fitting and removing roto-translational motion, blue VPS9^{WT}, red VPS9^{R1611W}.*

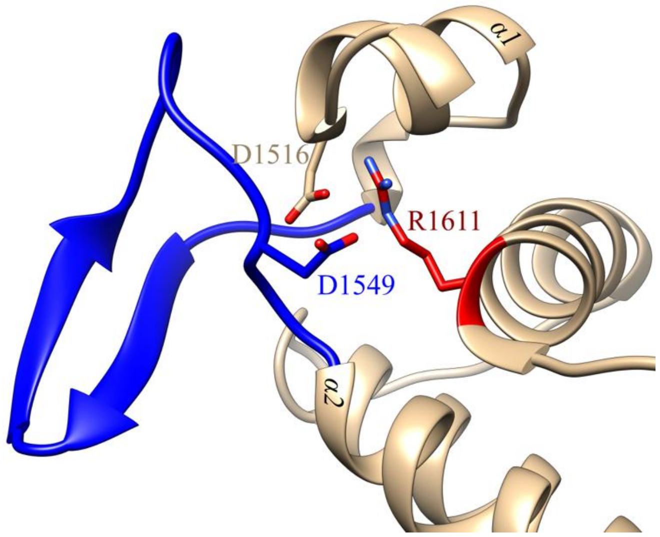

Figure S12. *VPS9 beta-strand region. The AlphaFold prediction of the VPS9WT domain suggest the existence of electrostatic interactions between R1611 and D1549, belonging to the coil-beta strand-coil portion of VPS9 between the alpha1 and alpha2 helices. The abolition of this interactions and the steric hindrance of the bulky apolar side chain of the tryptophane upon R1611W mutation may explain the infolding of the beta-strand.*

1.8 T-REMD temperature, validation and estimation of the error in the Free Energy Surface

Table S1. *Temperature of the parallel simulations for the REMD generated according to the van der Spooel algorithm*[5].

**REMD Temperatures**
298.15, 299.00, 299.84, 300.70, 301.55, 302.40, 303.26, 304.12, 304.98, 305.84, 306.71,
307.58, 308.44, 309.31, 310.19, 311.06, 311.94, 312.82, 313.70, 314.58, 315.46, 316.35,
317.24, 318.13, 319.02, 319.91, 320.81, 321.71, 322.61, 323.51, 324.41, 325.32, 326.22,
327.13, 328.05, 328.96, 329.88, 330.80, 331.72, 332.64, 333.57, 334.49, 335.42, 336.35,
337.28, 338.22, 339.15, 340.09, 341.03, 341.98, 342.92, 343.87, 344.82, 345.77, 346.72,
347.68, 348.64, 349.60, 350.56, 351.52, 352.49, 353.47, 354.44, 355.42, 356.39, 357.37,
358.35, 359.33, 360.32, 361.30, 362.29, 363.28, 364.28, 365.27, 366.27, 367.27, 368.27,
369.27, 370.28, 371.29, 372.30, 373.31, 374.32, 375.34, 376.36, 377.38, 378.41, 379.43,
380.46, 381.49, 382.53, 383.56, 384.60, 385.64, 386.69, 387.73, 388.78, 389.83, 390.88,
391.94, 392.99, 394.05, 395.12, 396.18, 397.25, 398.32, 399.39, 400.46, 401.54, 402.62,
403.70, 404.78, 405.87, 406.95, 408.05, 409.12, 410.22, 411.32, 412.42, 413.52, 414.63,
415.74, 416.85, 417.96, 419.08, 420.20, 421.32, 422.44, 423.57, 424.69, 425.83, 426.96,
428.09, 429.23, 430.38, 431.52, 432.67, 433.82, 434.97, 436.12, 437.28, 438.44, 439.60,
440.77, 441.93, 443.10, 444.28, 445.45, 446.63, 447.81, 448.99, 450.00

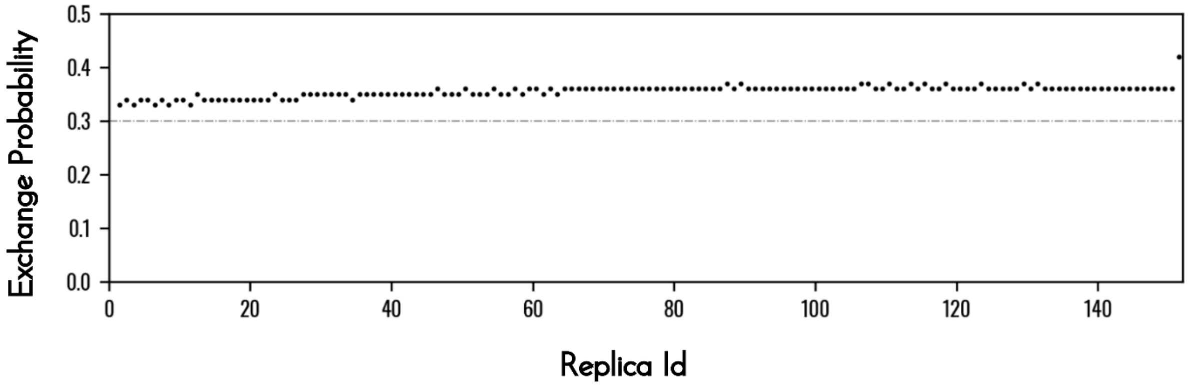

Figure S13. *Average exchange probability between replicas for VPS9^{WT}.*

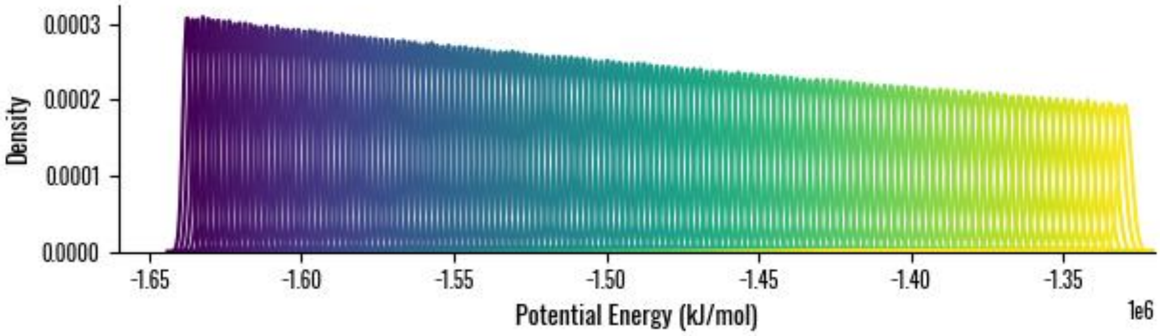

Figure S14*. Potential energy distribution for VPS9^{WT}.*

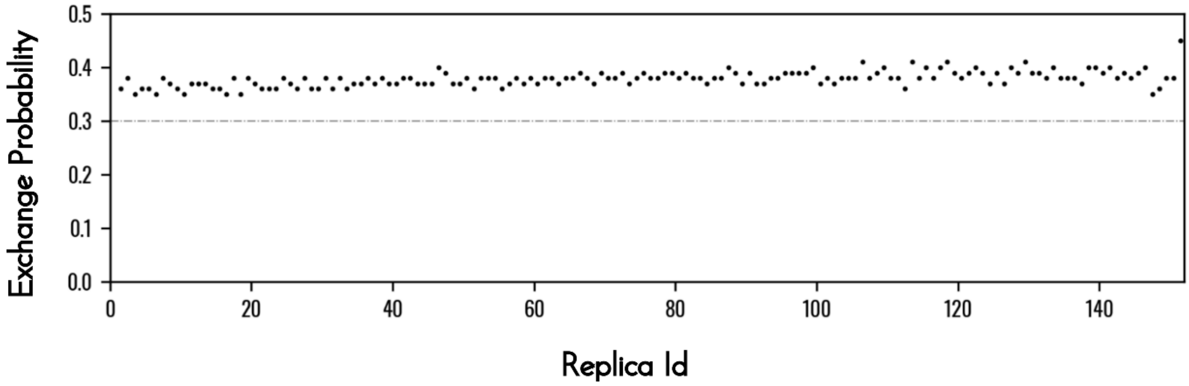

Figure S15. *Average exchange probability between replicas for VPS9^{R1611W}.*

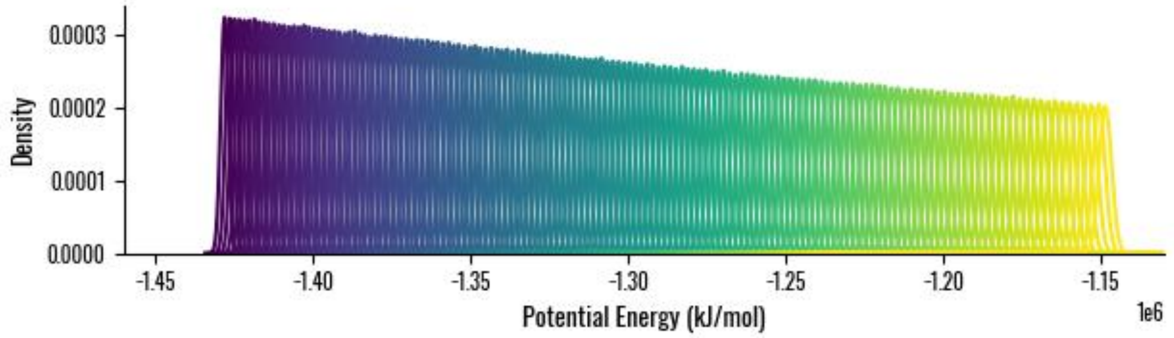

Figure S16. *Potential energy distribution for VPS9^{R1611W}.*

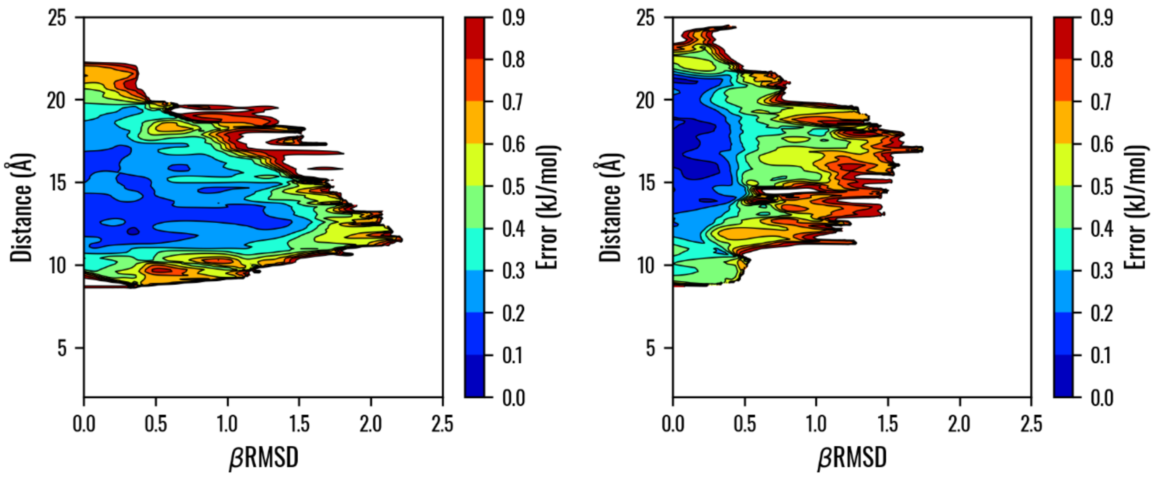

Figure S17. *Error on the estimation of the free energy profile for VPS9^{WT} (left) and VPS9^{R1611W} (right).*

2 Bibliography

(1) Wiederstein, M.; Sippl, M. J. ProSA-Web: Interactive Web Service for the Recognition of Errors in Three-Dimensional Structures of Proteins. *Nucleic Acids Research* **2007**, *35* (Web Server), W407–W410. https://doi.org/10.1093/nar/gkm290.

(2) ULC, C. C. G. Molecular Operating Environment (MOE), 2019.

(3) Uejima, T.; Ihara, K.; Goh, T.; Ito, E.; Sunada, M.; Ueda, T.; Nakano, A.; Wakatsuki, S. GDP-Bound and Nucleotide-Free Intermediates of the Guanine Nucleotide Exchange in the Rab5·Vps9 System. *Journal of Biological Chemistry* **2010**, *285* (47), 36689–36697. https://doi.org/10.1074/jbc.M110.152132.

(4) Delprato, A.; Lambright, D. G. Structural Basis for Rab GTPase Activation by VPS9 Domain Exchange Factors. *Nature Structural & Molecular Biology* **2007**, *14* (5), 406–412. https://doi.org/10.1038/nsmb1232.

(5) Patriksson, A.; Van Der Spoel, D. A Temperature Predictor for Parallel Tempering Simulations. *Phys. Chem. Chem. Phys.* **2008**, *10* (15), 2073. https://doi.org/10.1039/b716554d.
